# Novel Coumarins Derivatives for *A. baumannii* Lung Infection Developed by High-Throughput Screening and Reinforcement Learning

**DOI:** 10.1007/s11481-024-10134-w

**Published:** 2024-06-18

**Authors:** Jing Li, Zhou Lu, Liuchang Wang, Huiqing Shi, Bixin Chu, Yingwei Qu, Zichen Ye, Di Qu

**Affiliations:** 1 The Key Laboratory for Surface Engineering and Remanufacturing in Shaanxi Province, Key Laboratory of Chemistry of New Material of Functional Inorganic Composites, School of Chemical Engineering, Xi’an University, Xi’an, Shanxi China; 2 Department of Health Service, Medical Service Training Base, The Fourth Military Medical University, Xi’an, Shanxi China; 3 Department of Clinical Pharmacy, General Hospital of Western Theater Command, Chengdu, Sichuan China; 4 Department of Burn and Plastic Surgery, Zibo Prevention and Treatment Hospital for Occupation Diseases, Zibo, Shandong China; 5 Pancreatic Injury and Repair Key Laboratory of Sichuan Province, The General Hospital of Western Theater Command, Chengdu, Sichuan China; 6 Department of Cardiology, Xijing Hospital, The Fourth Military Medical University, Xi’an, Shanxi China

**Keywords:** *A. baumannii*, Coumarins, Lung infection, High-throughput screening, Reinforcement learning, Structure optimization

## Abstract

With the increasing resistance of *Acinetobacter baumannii* (*A. baumannii*) to antibiotics, researchers have turned their attention to the development of new antimicrobial agents. Among them, coumarin-based heterocycles have attracted much attention due to their unique biological activities, especially in the field of antibacterial infection. In this study, a series of coumarin derivatives were synthesized and screened for their bactericidal activities (Ren et al. 2018; Salehian et al. 2021). The inhibitory activities of these compounds on bacterial strains were evaluated, and the related mechanism of the new compounds was explored. Firstly, the MIC values and bacterial growth curves were measured after compound treatment to evaluate the antibacterial activity in vitro. Then, the in vivo antibacterial activities of the new compounds were assessed on *A. baumannii*-infected mice by determining the mice survival rates, counting bacterial CFU numbers, measuring inflammatory cytokine levels, and histopathology analysis. In addition, the ROS levels in the bacterial cells were measured with DCFH-DA detection kit. Furthermore, the potential target and detailed mechanism of the new compounds during infection disease therapy were predicted and evidenced with molecular docking. After that, ADMET characteristic prediction was completed, and novel, synthesizable, drug-effective molecules were optimized with reinforcement learning study based on the probed compound as a training template. The interaction between the selected structures and target proteins was further evidenced with molecular docking. This series of innovative studies provides important theoretical and experimental data for the development of new anti-*A. baumannii* infection drugs.

## Introduction

*Acinetobacter Bowman* (*A. baumannii*) is an opportunistic gram-negative bacterium with highly resistant to conventional prescription antibiotics and has excellent viability under various environmental conditions (Ayoub Moubareck et al. 2020; Jean et al. 2022). *A. baumannii* is one of the most important clinical pathogens with increasing isolation rate, infection rate and drug resistance, which has become a challenge in the global anti-infection field (Mea et al. 2021; Oliveira et al. 2020). *A. baumannii* now accounts for nearly 12% of all hospital-acquired infections worldwide and up to one-third of hospital-acquired infections in Asian countries (Gandra et al. 2020). It is also the most important “Super-bacterium” in our country and one of the most important pathogens of nosocomial infection. But there is no vaccine to prevent *A. baumannii* infection in the world, we need develop new weapon for the *A. baumannii* infection therapy with excellent pharmacological effect and low toxicity.

Studies indicated that designing novel anti-bacterial compound with new structure and mechanism may be a good choice for overcome antibiotic resistance in *A. baumannii*. Coumarin, also known as bisfuran cyclooxaphthalone, is a kind of lactone compound widely present in nature, mostly in *Rutaceae* and *Umbelliferae*, which showed different biological properties, such as antimicrobial, antibacterial, antifungal, antioxidant, antitumor, anticancer, antiviral, anti-inflammatory, and so on (Bhattarai et al. 2021; Feng et al. 2020; G et al. 2022). In our previous research, a coumarin derivative DCH has been proved with excellent anti-bacteria effect through binding with the ArgR regulator in MRSA (Qu et al. 2020). Thus, more novel structures were designed for the anti-*A. baumannii* evaluation in this study. The computer reinforcement learning methods were popular in the novel compounds development, which could predicate more effective candidate compounds via the known structures, and give a preliminary forecast about the biological activity of synthesized compounds. Thus, in this research, after the compound activity screening, target docking and ADMET predication, more novel derivatives were designed with computer for further anti-*A. baumannii* evaluation.

In this present research, coumarin derivatives with novel structure were designed and synthesized. Molecule and supramolecular structures of the compounds were identified and then the biological activities were evaluated. The MIC values showed the coumarin derivatives (1–5) have stronger anti-bacterial activity than the derivatives (6–10). Among these compounds, compound **1** (named as S1032 in the biological experiments) exhibited most excellent inhibitory activity on the *A. baumannii* strain. Additional, the survival rate of the *A. baumannii* infected mice was also up-regulated by the S1032 dose dependently, and the *A. baumannii* CFU numbers in lung tissue was down-regulated as well, suggesting the protective activity of the S1032 in vivo. Besides, the S1032 could also reduce the releasing of inflammatory cytokines and tissue damage of the mice. For the mechanism exploration, docking were carried out and he OmpA, BaeSR, AdeSR, AroA and CsuE proteins were predicated could formation covalent binding with S1032, which explained the excellent inhibitory activity on the multiplication, growth and biofilm formation of *A. baumannii*. All the results above provided the basis and new strategy for developing new anti-*A. baumannii* infection agents. Moreover, based on our previous study of using reinforcement learning technic for generating biologically effective drug molecules (Li et al. 2022), S1032 has been used as training template for conducting the reinforcement learning study, with the aim to optimize potential drug molecules that may also interfere with multiplication, growth and biofilm formation of *A. baumannii*, which could be the following research direction.

## Methods

### Synthesis and Characterization of Coumarins 1–10

**Scheme 1:**
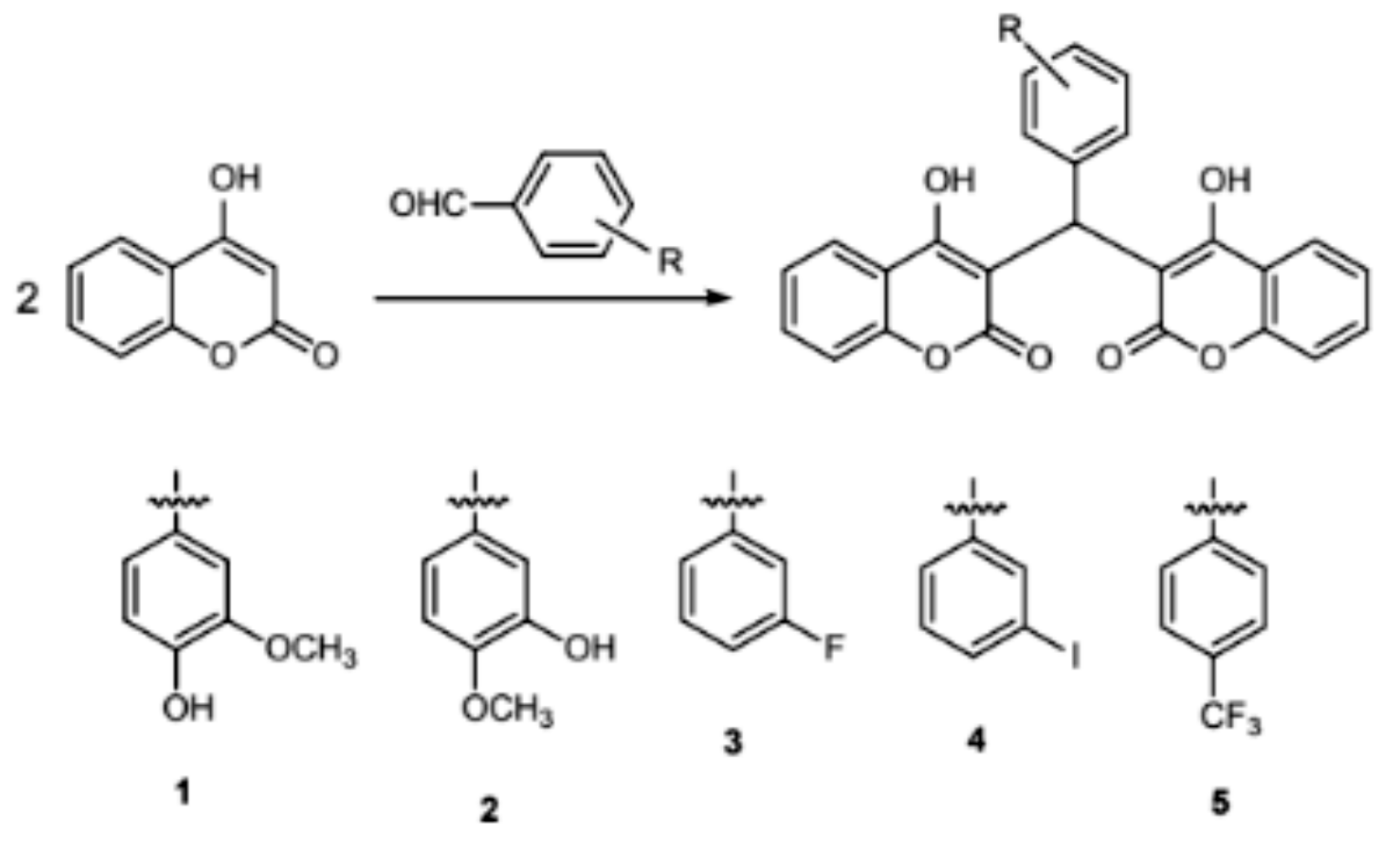
Synthetic route of compounds 1–5

Coumarins 1–5 were synthesized according to the methods of a previous report (Scheme 1) (Vega-Granados et al. 2021; Hersi et al. 2020). A mixture of aromatic aldehydes (10 mmol) and 4-hydroxycoumarin (20 mmol) was dissolved in 100 mL of EtOH. A few drops of piperidine were added, and the mixture was stirred for 3 h at room temperature. After reaction completion as determined by TLC, water was added until precipitation occurred. The solid was filtered off and then recrystallized from ethanol to give compounds 1–5.

3,3’-((4-hydroxy-3-methoxyphenyl)methylene)bis(4-hydroxy-2*H*-chromen-2-one) (**1**): m.p. 209–210 °C. IR (KBr pellet cm^− 1^): 2920, 1650, 1610, 1520, 1412, 1340, 1102, 761 cm. ^1^H NMR (CDCl_3_, δ, ppm): 11.581(s, 1H), 11.286(s, 1H), 8.021–8.053(t, 2 H), 7.606–7.649(m, 2 H), 7.398–7.419(d, 4 H), 6.852–6.873(d, 1H), 6.680–6.738(m, 2 H), 6.064(s, 1H), 5.579(s, 1H), 3.748(s, 3 H). HRMS (ESI+): m/z: calcd for C_26_H_18_O_8_: 481.0894 [M + Na]+; found: 481.0812.

3,3’-((3-hydroxy-4-methoxyphenyl)methylene)bis(4-hydroxy-2*H*-chromen-2-one) (**2**): m.p. 210–211 °C. IR (KBr pellet cm^− 1^): 2910, 1650, 1630, 1510, 1345, 1320, 1024, 761 cm. ^1^H NMR (CDCl_3_, δ, ppm): 11.612(s, 1H), 11.277(s, 1H), 8.034–8.070(d, 2 H), 7.623–7.666(m, 2 H), 7.414–7.435(d, 4 H), 6.799–6.819(t, 2 H), 6.708–6.729(t, 1H), 6.047(s, 1H), 5.612(s, 1H), 3.895(s, 3 H). HRMS (ESI+): m/z: calcd for C_26_H_18_O_8_: 481.0894 [M + Na]+; found: 481.0876.

3,3’-((3-fluorophenyl)methylene)bis(4-hydroxy-2*H*-chromen-2-one) (**3**): m.p. 221–223 °C. IR (KBr pellet cm^− 1^): 2915, 1721, 1630, 1520, 1422, 1323, 1012, 761 cm. ^1^H NMR (CDCl_3_, δ, ppm): 11.616(s, 1H), 11.327(s, 1H), 8.023-8. 109(q, 2 H), 7.645–7.688(m, 2 H), 7.435-7. 456(d, 4 H), 7.301–7.340(m, 1H), 6.941–7.047(m, 3 H), 6.095(s, 1H). HRMS (ESI+): m/z: calcd for C_25_H_15_FO_6_: 453.0745 [M + Na]+; found: 453.0751.

3,3’-((3-iodophenyl)methylene)bis(4-hydroxy-2*H*-chromen-2-one) (**4**): m.p. 231–232 °C. IR (KBr pellet cm^− 1^): 2932, 1721, 1620, 1571, 1432, 1310, 1021, 761 cm. ^1^H NMR (CDCl_3_, δ, ppm): 11.576(s, 1H), 11.308(s, 1H), 8.028-8. 109(q, 2 H), 7.628–7.691(m, 3 H), 7.547–7.550(d, 1H), 7.397–7.457(q, 4 H), 7.215–7.235(d, 1H), 7.064–7.103(t, 1H), 6.074(s, 1H). HRMS (ESI+): m/z: calcd for C_25_H_15_IO_6_: 560.9806 [M + Na]+; found: 560.9828.

3,3’-((4-(trifluoromethyl)phenyl)methylene)bis(4-hydroxy-2*H*-chromen-2-one) (**5**): m.p. 221–222 °C. IR (KBr pellet cm^− 1^): 2930, 1712, 1634, 1560, 1330, 1310, 1014, 763 cm. ^1^H NMR (DMSO-*d*_6_, δ, ppm): 8.419–8.439(d, 2 H), 7.770–7.809(q, 2 H), 6.626–7.677(q, 4 H), 7.513–7.583(m, 4 H), 4.985(s, 1H). HRMS (ESI+): m/z: calcd for C_26_H_15_F_3_O_6_: 503.0713 [M + Na]+; found: 503.0731.

**Scheme 2:**
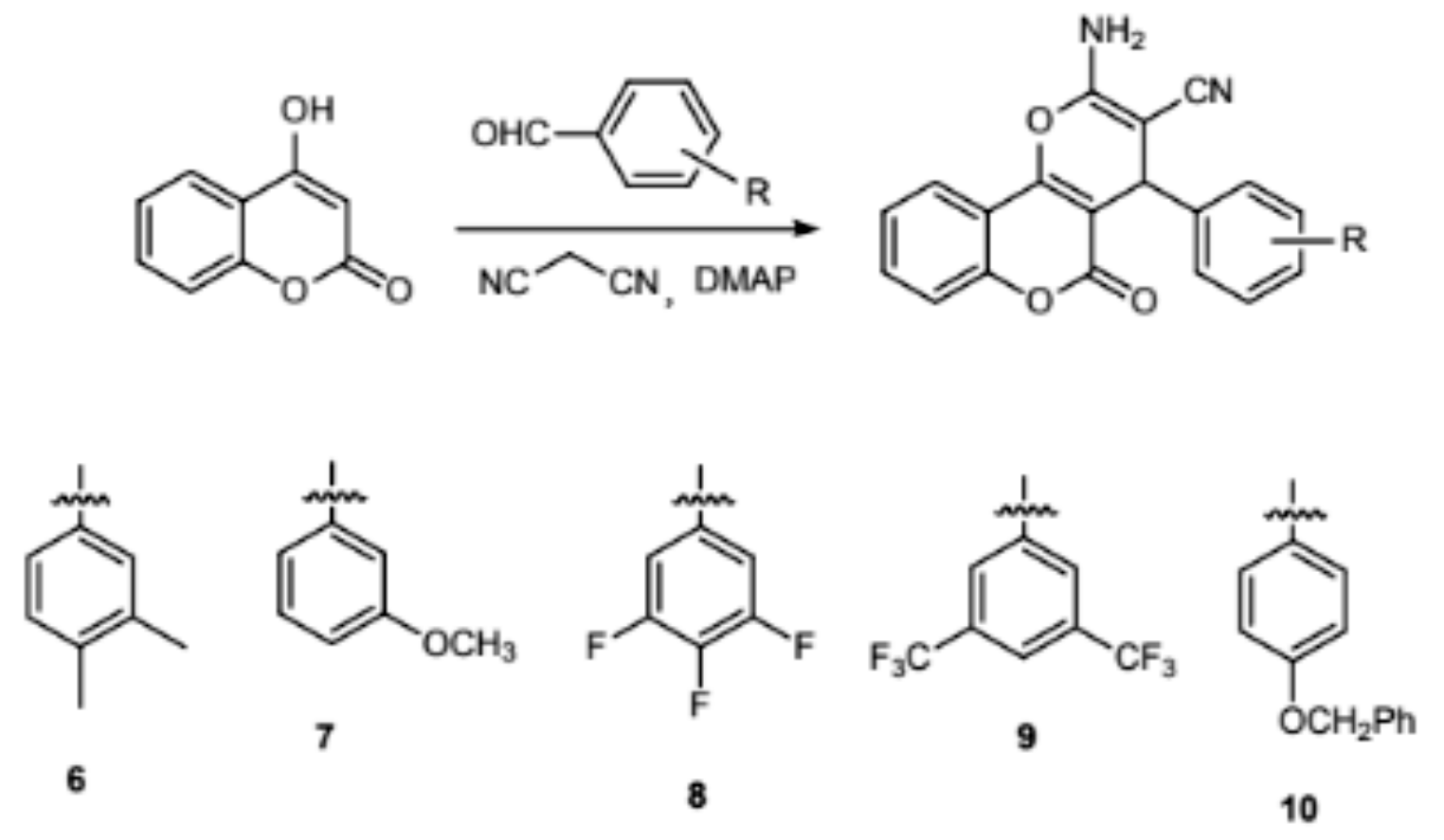
Synthetic route of compounds 6–10

Coumarins (6–10) were also synthesized according to a reported procedure (Scheme 2) (Li et al. 2022). A mixture of 4-hydroxycoumarin (10 mmol), aromatic aldehydes (10 mmol), malononitrile (10 mmol) and 4-(dimethylamino)pyridine (DMAP) (1 mmol) in ethanol (100 mL) was refluxed for 2–3 h and then cooled to room temperature. The solid was filtered off and then recrystallized from ethanol to give compounds 6–10.

2-amino-4-(3,4-dimethylphenyl)-5-oxo-4*H*,5*H*-pyrano[3,2-*c*]chromene-3-carbonitrile (6): m.p. 232–233 °C. IR (KBr pellet cm^− 1^): 2970, 1821, 1745, 1612, 1400, 1310, 1014, 756 cm. ^1^H NMR (DMSO-*d*_6_, δ, ppm): 7.887–7.910 (q, 1H), 7.694–7.737 (m, 1H), 7.457–7.514 (q, 2 H), 7.379 (s, 2 H), 6.846–6.893 (q, 2 H), 6.731–6.757 (q, 1H), 4,411 (s, 1H), 3.714–3.717 (d, 6 H). HRMS (ESI+): m/z: calcd for C_21_H_16_N_2_O_3_: 367.1053 [M + Na]+; found: 367.1099.

2-amino-4-(3-methoxyphenyl)-5-oxo-4*H*,5*H*-pyrano[3,2-*c*]chromene-3-carbonitrile (7): m.p. 223–224 °C. IR (KBr pellet cm^− 1^): 2940, 1712, 1630, 1520, 1432, 1320, 1024, 761 cm. ^1^H NMR (DMSO-*d*_6_, δ, ppm): 7.890–7.913 (q, 1H), 7.697–7.736 (q, 1H), 7.424–7.516 (m, 4 H), 7.219–7.259 (t, 1H), 6.800-6.841 (q, 3 H), 4.432 (s, 1H), 3.726 (s, 3 H). HRMS (ESI+): m/z: calcd for C_20_H_14_N_2_O_4_: 369.0846 [M + Na]+; found: 369.0822.

2-amino-5-oxo-4-(3,4,5-trifluorophenyl)-4*H*,5*H*-pyrano[3,2-*c*]chromene-3-carbonitrile (8): m.p. 254–255 °C. IR (KBr pellet cm^− 1^): 2840, 1652, 1623, 1532, 1412, 1305, 1024, 761 cm. ^1^H NMR (DMSO-*d*_6_, δ, ppm): 7.884–7.907 (q, 1H), 7.709–7.753 (m, 1H), 7.463–7.520 (q, 4 H), 7.332–7.371 (q, 2 H), 4.569 (s, 1H). HRMS (ESI+): m/z: calcd for C_19_H_9_F_3_N_2_O_3_: 393.0457 [M + Na]+; found: 393.0421.

2-amino-4-(3,5-bis(trifluoromethyl)phenyl)-5-oxo-4*H*,5*H*-pyrano[3,2-*c*]chromene-3-carbonitrile (9): m.p. 229–230 °C. IR (KBr pellet cm^− 1^): 2934, 1734, 1633, 1554, 1332, 1243, 1014, 760 cm. ^1^H NMR (DMSO-*d*_6_, δ, ppm): 8.075 (s, 2 H), 8.017 (s, 1H), 7.902–7.925 (q, 1H), 7.717–7.760 (m, 1H), 7.577 (s, 2 H), 7.468–7.535 (m, 2 H), 4.868 (s, 1H). HRMS (ESI+): m/z: calcd for C_21_H_10_F_6_N_2_O_3_: 475.0488 [M + Na]+; found: 475.0460.

2-amino-4-(4-(benzyloxy)phenyl)-5-oxo-4*H*,5*H*-pyrano[3,2-*c*]chromene-3-carbonitrile (10): m.p. 265–267 °C. IR (KBr pellet cm^− 1^): 2828, 1643, 1620, 1532, 1324, 1245, 1014, 760 cm. ^1^H NMR (DMSO-*d*_6_, δ, ppm): 7.879–7.899 (d, 1H), 7.707–7.747 (t, 1H), 7.451–7.519 (m, 4 H), 7.300-7.392 (m, 3 H), 7.112–7.149 (t, 1H), 6.978–7.041 (m, 5 H), 6.821–6.841 (d, 2 H), 4.477 (s, 1H). HRMS (ESI+): m/z: calcd for C_26_H_18_N_2_O_4_: 445.1159 [M + Na]+; found: 445.1198.

### X-ray Crystallography

SuperNova diffractometer was utilized in acquiring XRD patterns. CrysAlisPro was employed to analyze intense data and converted the data to HKL files. SHELXS in the light of direct mean and SHELXL-2014 software based on least-squares strategy were employed respectively for the synthesis and refinement of original architectural modes. After the addition of non-H atoms, the anisotropic parameters could be mixed. Eventually, the entire H atoms could be fixed on the C atoms that bridged with AFIX commands in geometry. The as-prepared compounds’ refinement details and crystallography parameters are displayed in Table 1.

**Table 1:** Refinements details and crystallography parameters of the as-prepared compounds

Identification code	1	6
Empirical formula	C_26_H_18_O_8_	C_23_H_22_N_2_O_4_
Formula weight	458.40	390.43
Temperature/K	298 (2)	298 (2)
Crystal system	Monoclinic	Triclinic
Space group	*P*2_1_/*n*	*P-1*
a/Å	13.2383 (15)	7.805 (3)
b/Å	10.7719 (15)	11.986 (4)
c/Å	15.0137 (15)	12.438 (5)
α/°	90	109.344 (6)
β/°	95.810 (11)	103.882 (7)
γ/°	90	102.427 (7)
Volume/Å^3^	2130.0 (4)	1009.1 (7)
Z	4	2
D_calc_/g/cm^3^	1.429	1.285
µ/mm^− 1^	0.11	0.09
Goodness-of-fit on F^2^	1.028	1.029
Final R indexes [I > = 2σ (I)]	0.1051(2277)	0.0606(1861)
Final R indexes [all data]	0.3495(3470)	0.1526(3535)

### Strains and Animals

Methicillin-resistant *Staphylococcus aureus* N315 (MRSA N315); *Staphylococcus aureus* ATCC 29,213 (*S. aureus* ATCC 29,213); *Pseudomonas aeruginosa* (*P. aeruginosa*); *Klebsiella pneumonia* (*K. pneumoniae*); *Acinetobacter baumannii* (*A. baumannii*) and *Escherichia coli* (*E. coli*) were purchased from the American Type Culture Collection (ATCC). All the clinical *A. baumannii* strains were provided by the Zibo Prevention and Treatment Hospital for Occupation Diseases (Zibo, China). The bacteria were cultured on Nutrient agar plates (Sigma-Aldrich, USA) at 37℃ in an incubator and then transferred into Nutrient broth for strain replication at the condition of 220 rpm, 37℃.

BALB/c mice (5-6weeks, 20–22 g) were used in this research, which were purchased form the Animal Research Center of the Fourth Military Medical University (Xi’an, China). All the mice were maintained at a specific pathogen-free (SPF) environment of 20–25℃, combined with a 12- hour light/dark schedule and free standard laboratory mouse chow and water supply. All the preformation in mice were approved by the Institutional Animal Care and Use Committee (IACUC) of the Fourth Military Medical University, and conducted totally under the guidance of the health guidelines for the use of laboratory animals.

### MIC Determination

The minimum inhibitory concentration (MIC) values of the synthesized compounds were determined with broth micro-dilution technique for the anti-bacterial activity evaluation. This preformation was conducted strictly under the guidance of the instructions with some modifications (Kowalska-Krochmal and Dudek-Wicher 2021). In brief, the bacterial strains were cultured in Nutrient broth and seeded into the microtiter plates at a final concentration of 1 × 10^8^ CFU/ml overnight. Then, the synthesized compounds and reference antibiotic in 100 µl culture medium was added into the wells with serial concentrations (2–256 µg/mL) for another 24 h. The OD_600_ values of each well were measured, and the MIC values of the compounds were regarded as the lowest concentrations completely inhibit the bacterial growth.

### *A. baumannii* Growth Curves

The inhibitory activity of the synthesized S1032 on *A. baumannii* growth was evaluated in this experiment under the guidance of the following descriptions. The *A. baumannii* strain was collected and cultured in Nutrient broth at the condition of 220 rpm, 37 °C. Then the bacterial cells were planted into microtiter plates (1 × 10^8^ CFU/ml) with S1032 added for treatment, the same concentration of Ofloxacin and Gentamicin were used as control. The plates were placed in an automated Bioscreen C system in the automatic bacterial growth curve analyzer (Bioscreen, Finland) at 37 °C, and the absorbances of all the wells were determined every hour at 600 nm (Rivani et al. 2022).

### qPCR Assay

The qPCR was performed in this research to measure the relative expression of the genes related with bacterial biofilm formation. In brief, the *A. baumannii* bacterial cells were grown in Nutrient broth at 37 ℃ to exponential phase and then treated with the S1032 compound with the indicated concentrations for 2 h. After the indicated treatment, the *A. baumannii* bacterial cells were collected, washed, and the total RNA in the bacterial cells was extracted with TRIZOL reagent. After measuring the quality and quantity of the total RNA, which was then reverse transcripted into cDNA with reverse transcription kit (Qiagen) in 20 µl total reaction system. Finality the relative expression of biofilm formation related genes (*bfmR*, *bfmS*, *CsuA*, *CsuB*) were determined by SYBR Green Master Mix after S1032 treatment, *16s* was used as internal control. 2^−ΔΔCt^ method was used for statistical analysis. This research was performed three times. All the primer sequences were listed in Table 2.

**Table 2:** Sequences of primers used in this research

Primers	Sequences (5′-3′)
*bfmR F*	GTTTAACCGTTTGTCGTG
*bfmR R*	GTGGTTGAACTGGTTTCG
*BfmS F*	ACCGCCCGTAATCCGAAC
*BfmS R*	TGAACTTATTCCACCGCCTTTA
*CsuA F* ^[17]^	CAACGCTATGTGCTGCTGG
*CsuA R*	GGCCCACCCAAAGTAATCC
*CsuB F*	TATGCAGCAGATCCTCAG
*CsuB R*	TAAACTTTCCGTACAACG
*16s F* ^[18]^	CTCCTACGGGAGGCAGCAGT
*16s R*	TATTACCG CGGCTGCTGGC

### Mice Lung Infection Model

BALB/c mice (5-6weeks, 20–22 g) used in this present study were divided randomly into different groups: the negative control group (*n* = 7), positive control group (receive antibiotic treatment, *n* = 7) and S1032 treatment groups (1, 2 and 5 mg/kg, *n* = 7). Then the mice were challenged with 5 × 10^9^ CFU *A. baumannii* in 20 µl Nutrient broth via nasal drops. After bacterial infection, S1032 (1, 2 and 5 mg/kg) and Ofloxacin (5 mg/kg) was given for 7 consecutive days, with saline used as negative control. The survival rates of the mice in each group were monitored since infection, and the cumulative percentage survival was plotted (Tansho-Nagakawa et al. 2021).

### Therapeutic Activity Evaluation

To evaluate the therapeutic activity of the synthesized S1032, the *A. baumannii* bacterial CFU numbers in lung tissue was determined in this research. Briefly, after the construction of lung infection mice model, S1032 was given for treatment with indicated concentrations (1, 2 and 5 mg/kg) for three consecutive days. 7 days after infection, the animals were euthanized for the infected tissue collection. The tissue samples were grinded, diluted and dripped onto Nutrient agar plates for 24 h cultivation at 37℃ and 5%CO_2_ condition. The numbers of bacterial colony were counted and recorded. This experiment was repeated at least three times, and the results were presented as mean ± SD.

After the collection of infected mice lung tissue, which were then fixed in 10% neutral buffered formalin for 24 h. Then the tissue samples were dehydrated and processed into paraffin sections totally according to the standard procedure. The paraffin sections were then stained with hematoxylin and eosin (H&E) staining for histopathology analysis, and the microscope was used for observation of tissues morphologies.

### Biofilm Formation Assay

To evaluate the inhibitory activity of the S1032 compound on bacterial biofilm formation, the crystal violet staining assay was performed in this research. All the conduction was finished strictly under the guidance with only a little modification (Khan et al. 2022). In short, *A. baumannii* strain were seeded in 96 well plates and cultured in the incubator at the condition of 37℃ for 24 h. After the bacterial biofilm formation, 0.1% crystal violet (CV) (Sigma) solution was added into wells for staining at 37 °C for 10 min. After that, PBS was used to wash the unattached CV and 95% ethanol was added to detainee the stained biofilm cells. The solution absorption was measured at 600 nm, each group has independent replicates, and this experiment was repeated at least three times.

### Scanning Electron Microscope for Biofilm Observation

The bacterial culture solution was diluted to 1 × 10^8^CFU/ml. 500 µl of the diluted bacterial solution was added to the 24-well cell culture plate wells that contained sterilized coverslips. The plate was incubated at 37℃ in a incubator for 36 h. The coverslips with adhered bacteria were gently washed with sterile PBS to remove any unattached free-floating bacteria. Then, 2.5% (v/v) glutaraldehyde solution was added, and the samples were fixed overnight at 4℃. The fixative was aspirated, and the samples were washed three times with sterile PBS, each time for 10 min. After washing with sterile water several times, the samples were dehydrated with 30%, 50%, 70%, and 90% ethanol for 15 min each, and finally dehydrated twice with 100% ethanol, each time for 15 min. The samples were then placed in a vacuum freeze-drying machine and freeze-dried for 2–3 h. The dried samples were taken out, attached to a sample holder with conductive adhesive, and observed under a scanning electron microscope (Gould et al. 2022).

### Intracellular ROS Determination

The *A. baumannii* bacterial cells were grown in Nutrient broth at 37 ℃ to exponential phase and seeded into the microtiter plates at the final destiny of 5 × 10^7^ CFU/ml overnight. Then, the Nutrient broth containing the active S1032 with the final concentrations of 0.25, 0.5 and 1 µg/m was added into the bacterial wells for 2 hours incubation. After that, the *A. baumannii* bacterial cells were collected, washed, centrifuged and re-suspended with PBS solution. 2’,7’-dichlorodihydrofluorescein diacetate (DCFH-DA, 10 µl) was added to the above suspension to detect the content of overall intracellular ROS. After incubation 30 min in the dark, the bacteria cells were harvested, washed at least three times to remove the extra fluorescence probe. Fluorescence intensity of each sample was detected at an excitation wavelength of 484 nm and an emission wavelength of 525 nm (Wang et al. 2022). This research was repeated at least three times.

### Cytotoxicity Evaluation

Cell Counting Kit-8 (CCK-8) assay was conducted in this research to evaluate the cytotoxicity of active S1032 against human umbilical vein endothelial cells (HUVEC) and human embryonic kidney (Hek) 293T cells. This research was conducted under the guidance of manufactures’ instruction. The HUVEC and 293T cells were collected and seeded into the 96 well plates at the destiny of 5000 cell/well. After the cell got the 70–80% confluence, the S1032 was added for 48 h incubation with serial different concentrations (2.5, 5, 10, 20, 40, 80, 100 and 200 µM). After that, the culture medium was discarded, and the cells were washed with PBS solution. Fresh culture medium containing 10 µL CCK-8 reagents was added into the well for 4 h incubation. Then, the absorbance of each well was measured at 490 mm. This preformation was conducted at least three times, and the results were presented as mean ± SD.

### ADMET Predication Online

The ADMET parameter of the S1032 was predicated using SWISS ADME online tool (http://www.swissadme.ch/) (Daina et al. 2017). The SMILES of the compound were produced and was lodged into the SWISS ADME website, and the physicochemical properties, lipophilicity, water solubility, pharmacokinetics, druglikeness and medicinal chemistry was predicated.

### Molecular Docking

Four structures were considered as the target proteins for the docking studies, their structures were retrieved from Protein Data Bank (PDB) and the PDB IDs were 3TD3 (Park et al. 2012), 4JAS (Podgornaia et al. 2013), 5BUF (Sutton et al. 2016) and 6FJY (Pakharukova et al. 2018). The active sites of S1032 and predicated target proteins were measured with molecular docking study via AutoSite (v1.1) tool software (Ravindranath and Sanner 2016). Firstly, the 3D structures of target proteins in PDB format were downloaded from RCSB Protein Data Bank. Then, the ligand structure (S1032) was prepared by Avogadro (v1.2) (Hanwell et al. 2012), an energy minimization was performed through steepest descent algorithm adopting general Amber force field (GAFF) (Wang et al. 2004). After obtaining structures for both ligand and proteins, we used AutoDockTools (v1.5.7 patch 1) to prepare the docking simulations, and the docking studies were conducted using AutoDock4 (v4.2.6) (Morris et al. 2009). For the docking simulation, 50 potential docking poses are evaluated using the Lamarckian genetic algorithm (LGA). The binding affinity of the complexes was recorded with a unit of kcal/mol. The protein-ligand interactions were analyzed using PLIP (Adasme et al. 2021).

### Reinforcement Learning Studies

The reinforcement learning approach that was implemented and performed in the current study is based on the widely used molecule deep Q-networks (MolDQN) (Zhou et al. 2019). During the reinforcement learning process, a known molecule that has been proved to have excellent bioactivities depending on demands and requirements of the study (in the current work, the desired molecule is the inhibitor of *A. baumannii*) is used as the training template (Almihyawi et al. 2022; Abdelaziz et al. 2022). Then, multiple actions are allowed to modify the template and generate novel structure which is expected to have similar or even better bioactivity than the given template, the allowed actions are atom addition, bond addition, bond removal and un-modification. After a certain number of actions has been performed. Three qualification characteristics are determined upon the optimized structure, these qualification characteristics are: (i). binding affinity energy between the optimized molecule and the protein; (ii). synthetic accessibility (SA) score; (iii) quantitative estimate of drug-likeness (QED) score. Explicitly, the binding affinity energy is evaluated by QuickVina 2 (Alhossary et al. 2015), which has been proven to have both accuracy and efficiency as AutoDock 4, as has been mentioned in the above docking study, the gym-molecule library has been employed to calculate the SA score, and the open source library rdkit (https://www.rdkit.org/) has been utilized to estimate the QED score. For these three qualification measurements, the Smile code of the molecular structure has been used as input, consequently, the 3-dimensional structure is effected using Open Babel.

For each of the ligand (S1032) – protein (3TD3, 4JAS, 5BUF and 6FJY) combination, the maximum number of allowed modifications (actions) is set to 20, during the modification, only C, N, O atoms are allowed, and up to 10,000 molecule structures are evaluated. After the reinforcement learning study, four molecules that are showing excellent binding affinity energies, SA and QED scores are selected, and a further molecular docking simulation has been performed using the same procedure described in the above Molecular Docking section.

### Reinforcement Learning Studies

The reinforcement learning approach that was implemented and performed in the current study is based on the widely used molecule deep Q-networks (MolDQN) (Zhou et al. 2019). During the reinforcement learning process, a known molecule that has been proved to have excellent bioactivities depending on demands and requirements of the study (in the current work, the desired molecule is the inhibitor of *A. baumannii*) is used as the training template. Then, multiple actions are allowed to modify the template and generate novel structure which is expected to have similar or even better bioactivity than the given template, the allowed actions are atom addition, bond addition, bond removal and un-modification. After a certain number of actions has been performed. Three qualification characteristics are determined upon the optimized structure, these qualification characteristics are: (i). binding affinity energy between the optimized molecule and the protein receptor; (ii). synthetic accessibility (SA) score; (iii) quantitative estimate of drug-likeness (QED) score. Explicitly, the binding affinity energy is evaluated by QuickVina 2, which has been proven to have both accuracy and efficiency as AutoDock 4, as has been mentioned in the above docking study, the gym-molecule library has been employed to calculate the SA score, and the open source library rdkit (https://www.rdkit.org/) has been utilized to estimate the QED score. For these three qualification measurements, the Smile code of the molecular structure has been used as input, consequently, the 3-dimensional structure is effected using Open Babel.

For each of the ligand (S1032) – protein (3TD3, 4JAS, 5BUF and 6FJY) combination, the maximum number of allowed modifications (actions) is set to 20, during the modification, only C, N, O atoms are allowed, and up to 10,000 molecule structures are evaluated. After the reinforcement learning study, four molecules that are showing excellent binding affinity energies, SA and QED scores are selected, and a further molecular docking simulation has been performed using the same procedure described in the above Molecular Docking section.

### Statistical Analysis

All the biological experiments in this research were conducted in triplicate and the results were presented as the mean ± standard deviation (SD). SPSS 20.0 statistical software (SPSSInc., Chicago, IL, USA) was recommended for the analysis. Two-tailed Student’s *t*-test was used to analyze the difference between two groups, and the one-way ANOVA method was carried out for the analysis among more than three groups. Statistical significance was considered as *P* < 0.05.

### Ethics Statement

All the in vivo experiments were approved by the Committee of Animal Ethics of the Fourth Military Medical University (Xi’an, China). All the preformation was strictly in accordance with the manufactures’ instructions with some modifications.

## Results

### Molecular Structure of Novel Compounds

**Fig. 1:**
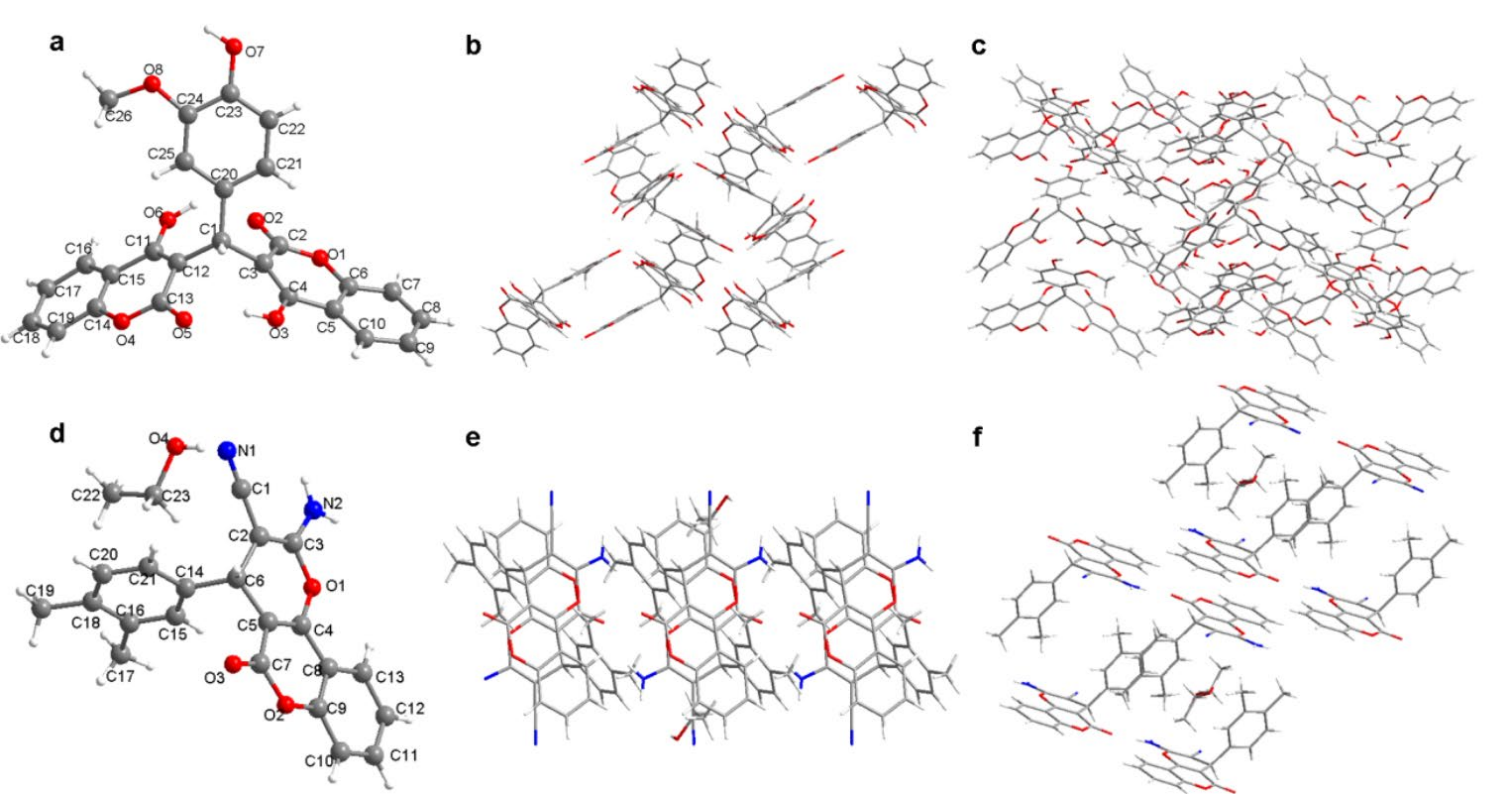
The molecule structure for **1** (a). The supramolecular structure of **1** (b-c). The molecule structure for **6** (d). The packing structure of **6** from different direction (e-f)

The crystal structure of 1 was identified by the single-crystal diffraction, data analysis reveal that 1 crystallizes in the monoclinic crystal system with the P2_1_/n space group. The molecular structural unit of 1 is displayed in Fig. 1a. The skeleton of 1 consists of three parts: two 4-hydroxy-2 H-chromen-2-one molecules [(C2-C10, O1-O3) and (C11-C19, O4-O6)]-A and one 4-hydroxy-3-methoxybenzaldehyde (C1, C20-C26, O7 and O8)-B. During the synthesis procedure, two A molecules and one B molecule were bind via the sp^3^ C1 atom of the B, giving the molecule structure of 1. And further the adjacent molecules were further packed via the intermolecular interaction to give a supramolecule network (Fig. 1b-c).

The x-ray single-crystal diffraction measurements were used to explore the micro-structure of 6, revealing that 6 shows the triclinic crystal system with P-1 space group. As shown in Fig. 1d, the molecule structure of 6 consist of two benzene ring and two six-membered rings. The benzene ring (C8-C13) connects the six-membered ring (C4, C5, C-C9 and O2) via the sharing of the bond C8-C9, and there exists O3 atom one the six-membered ring via the double bond of C7 = O3. The two six-membered rings (C4, C5, C-C9 and O2) and (C2-C6 and O1) were connected via the C4-C5 bond which give a three-ring structure. There exists one cyano (C1-N1) and one amino (N2H2) groups on the six-membered via the bonds of C1-C2 and N2-C3. In addition, the benzene ring (C14-C16, C18, C20, C21) connect the six-membered ring (C2-C6 and O1) via the bond of C14-C16. Also, there exist two methyl groups (C19H3 and C17H3) connect the benzene ring via the bond of C18-C19 and C16-C17. And further there exist one ethanol molecule in the structure of 6. In addition, the adjacent molecules were further connected to produce a dense packing structure (Fig. 1e-f).

### Potent Activity of Synthesized Compounds Against Bacteria In Vitro

**Fig. 2:**
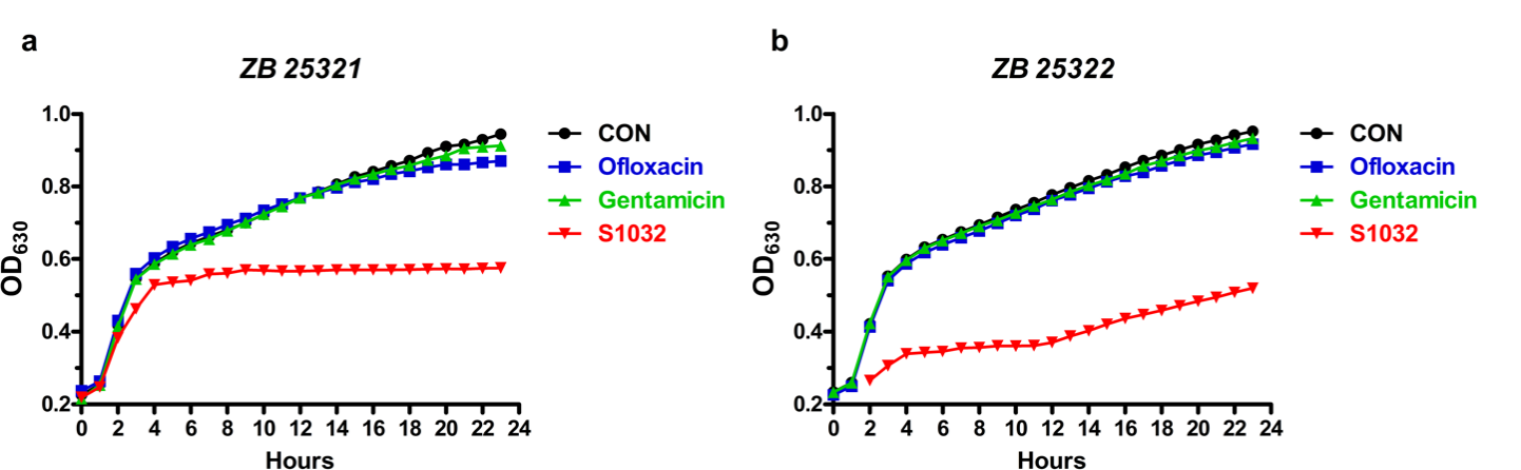
Inhibitory activity of S1032 against *A. baumannii* growth in vitro. Growth curve of *A. baumannii* ZB 25,321 after S1032 treatment **(a).** Growth curve of *A. baumannii* ZB 25,322 after S1032 treatment **(b)**

Two series of chemical compounds, coumarin derivatives (1–5) and derivatives (6–10) were designed and synthesized in this research to screen possible antibacterial small-molecular chemical compounds. The anti-bacterial activities of these new compounds were evaluated via MIC determination. As the results showed in Table 1, we can see the anti-bacterial activity of the coumarin derivatives (1–5) was much stronger than that of the derivatives (6–10). The coumarin derivatives 1 (named as S1032) exhibited most excellent biological effect on Gram negative bacteria, especially against *A. baumannii* with minimal inhibitory concentration (MIC) of 0.5 µg/ml. The S1032 also showed inhibitory effect on other Gram negative bacteria, such as *P.* aeruginosa, *K. pneumoniae*, *A. baumannii* and *E. coli*, with MIC of 2–4 µg/ml, respectively. Next, more clinical *A. baumannii* strains (ZB 25,321–25,327) were collected then the MIC of the S1032 against these strains were measured and showed in Table 3. Compared with the positive control antibiotics, Ofloxacin and Gentamicin, S1032 showed much more outstanding inhibitory activity. This result indicated the clinical *A. baumannii* strains are resistant to Ofloxacin and Gentamicin, but sensitive to S1032 compound. Besides, the influence of the S1032 on the growth curves of clinic s *A. baumannii* strains was further measured. The results in Fig. 2 suggested the S1032 significantly reduced the growth curves of these strains, and the inhibition of S1032 against MDR-AB clinic strains was stronger than Ofloxacin and Gentamicin. This result was consistence with the MIC results in Table 4.

**Table 3:** MIC values of new compounds on bacteria

Compounds	MRSA N315	*S. aureus 29,213*	*P. aeruginosa*	*K. pneumoniae*	*A. baumannii*	*E. coli*
1	16	8	2	4	0.5	2–4
2	32	16	16	16	8	8
3	16	16	16	16	16	16
4	64	64	64	64	64	128
5	> 256	> 256	> 256	> 256	128	128
6	128	128	128	128	128	> 256
7	32	32	16	16	32	32
8	64	64	128	64	64	32
9	128	64	128	128	128	128
10	> 256	> 256	> 256	> 256	> 256	> 256
Ofloxacin	16	2	4	4	> 256	8
Gentamicin	32	4	8	8	> 256	1

**Table 4:** MIC values of S1032 on clinical *A. baumannii* strains

Compounds	ZB 25,321	ZB 25,322	ZB 25,323	ZB 25,324	ZB 25,325	ZB 25,326	ZB 25,327
S1032	0.5	0.5	0.5	0.5	0.5	0.5	0.5
Ofloxacin	> 256	> 256	> 256	> 256	> 256	> 256	> 256
Gentamicin	> 256	> 256	> 256	> 256	> 256	> 256	> 256

### Protective Activity of S1032 on *A. baumannii* Infected Mice

**Fig. 3:**
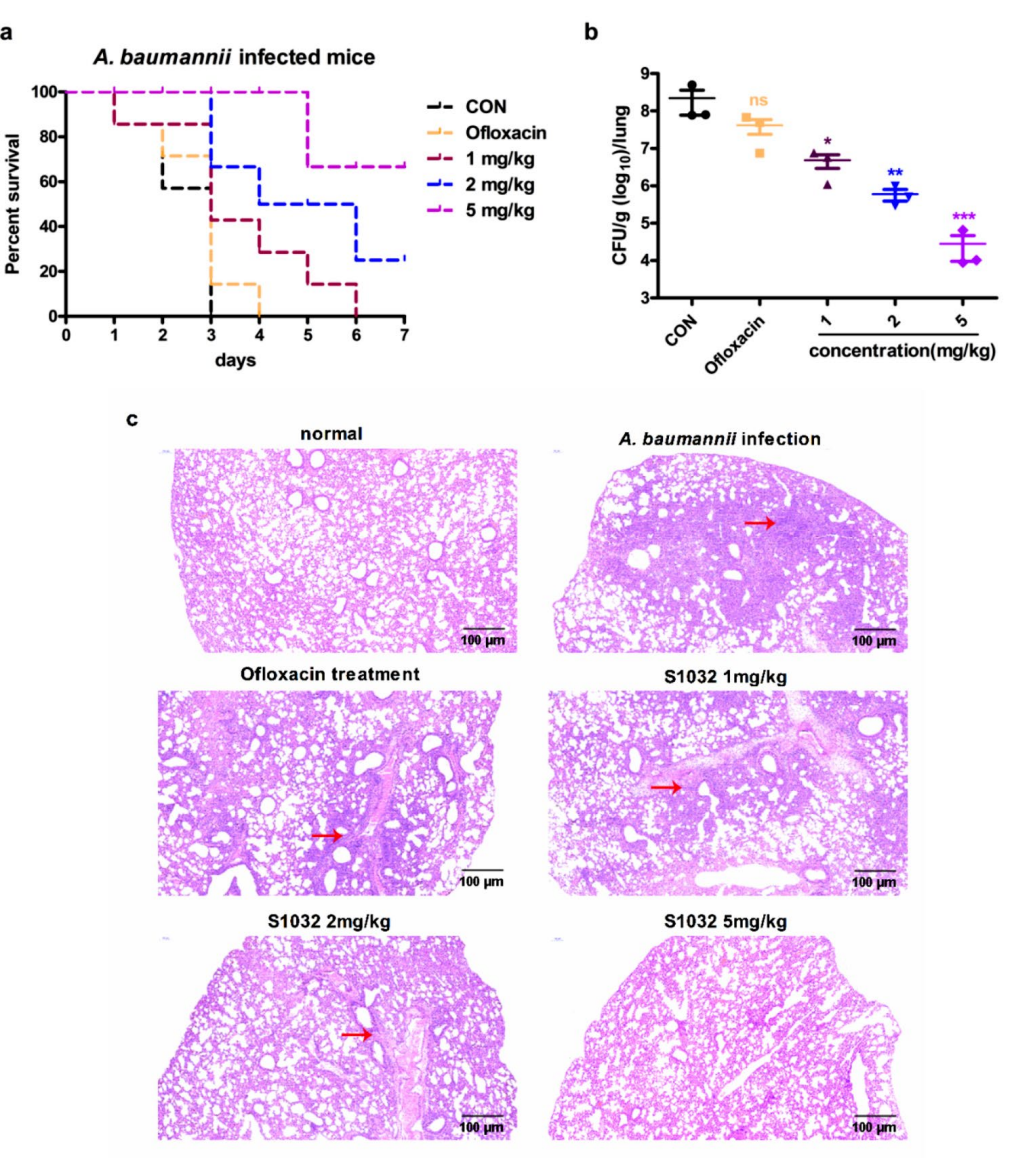
S1032 increased infected mice survival rate and reduced bacterial load. The *A. baumannii* was given to induce the infected animal model, followed by the S1032 treatment. The survival of the mice was observed once a day for 7 days since infection **(a)**. The bacterial load of lung tissue was determined with CFU assay **(b)**. The pathological damage of the lung organ was evaluated with H&E staining **(c)**

As the excellent inhibitory activity of the S1032 on MDR-AB clinic strains in vitro, ZB 25,321 was further used for bacterial infection mice model construction and in vivo therapeutic effect evaluation. S1032 was injected for treatment at 1, 2 and 5 mg/kg, Ofloxacin was used as control drugs. The results in Fig. 3a indicated that all the mice infected with the *A. baumannii* in the control group died within 3 days. The mice in the Ofloxacin treated groups also died within 3 days. While, after intraperitoneal administrated with the S1032 at the concentration of 1, 2 and 5 mg/kg, the death time of the mice was delayed, and the survival rate of the animal was up-regulated significantly. The survival rate was 85.7% under 5 mg/kg S1032 treatment (Fig. 3a). Furthermore, the *A. baumannii* CFU numbers in the infected ling tissue were also counted and the date suggested that the bacterial load in the model group was much higher than that of the control group. There was a significantly difference between these groups, with *P* < 0.005. Under the treatment of the S1032, the bacterial load in the lung was reduced obviously. The inhibition of the S1032 showed a dose dependent relationship, which is much stronger than the Ofloxacin at the concentration of 5 mg/kg (Fig. 3b). Hematoxylin and eosin (H&E) staining reveals that *A. baumannii* infection triggers a robust inflammatory response in the lungs, characterized by a substantial infiltration of inflammatory cells. Following the infection, lung tissue undergoes necrosis, which is evident through the deeper staining and the distinctive dark red hue, contrasting sharply with the normal surrounding tissue. Notably, infected tissue exhibits heightened congestion, clearly distinguishable from the uninfected control group. Strikingly, treatment with S1032 mitigates the inflammatory damage, necrosis, and local congestion in the lung tissue of mice. The salutary effect of S1032 is presumably achieved by impeding bacterial growth, thereby reducing tissue injury and ultimately enhancing the survival rates of infected mice. (Fig. 3c).

### Inhibition Effect of the S1032 on *A. Baumannii* Biofilm Formation

**Fig. 4:**
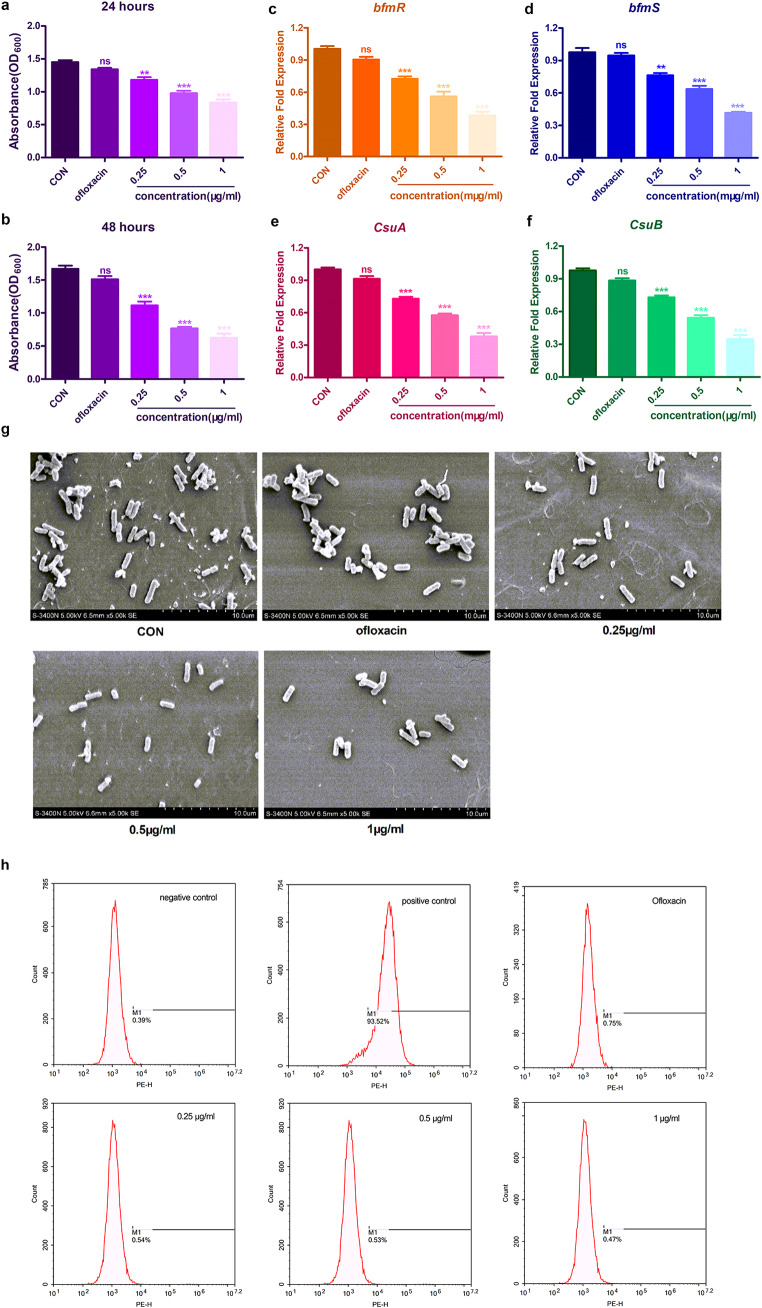
S1032 reduced *A. baumannii* biofilm formation. The *A. baumannii* were incubated in the plates for the biofilm formation, S1032 was added for treatment at 0.25, 0.5 and 1 µg/ml. The biofilm of the *A. baumannii* was labeled with 1% crystal violet staining assay **(a-b)**. The relative expression of the biofilm formation related genes (*bfmR*, *bfmS*, *CsuA*, *CsuB*) were measured with real time RT-CPR assay (c-f). The ROS accumulation in *A. baumannii* was measured with DCFH-DA detection kit in flow cytometer (g). *P < 0.05, **P < 0.01, and ***P < 0.001 vs. control, *n* = 5

During the infection procession, the *A. baumannii* has the capacity of the biofilm formation, which could hide themselves, escape antibiotics and finally lead to the biofilm-associated infections on indwelling medical devices (Daele et al. 1987). Thus, the suppression of the S1032 on the *A. baumannii* biofilm formation was evaluated in this study as well. The 1% crystal violet staining assay indicated that the biofilm of the *A. baumannii* could be significantly inhibited by the new compound at the sub-MIC with the concentration of 0.25, 0.5 and 1 µg/ml in a dose dependent manner (Fig. 4a-b). Next, the relative expression of the biofilm formation related genes was also determined with q-PCR. The results in biofilm formation suggested that the S1032 also showed excellent inhibitory effect on the biofilm formation genes expression with the sub-MIC concentrations dose dependently, and the scanning electron microscope (SEM) images also proved the inhibitory activity of the new S1032 against *A. baumannii* biofilm formation. (Fig. 4c-g).

The ROS in the bacterial cells was important for the bacteria survival and bacterial resistance. So, in this present research, the accumulation of ROS in *A. baumannii* after S1032 treatment was further determined with DCFH-DA detection kit. All the results were presented in Fig. 4h, we can see Ofloxacin has no influence on the ROS level in *A. baumannii*, which also proved the resistance of *A. baumannii* to Ofloxacin. Similar with Ofloxacin, the S1032 also showed almost no influence on the ROS levels in the bacterial cells. This result suggested that the anti-bacteria effect of S1032 was not mediated by the ROS accumulation in the bacterial cells.

### S1032 Targeted Several Proteins for the Anti-Bacteria Effect

**Fig. 5:**
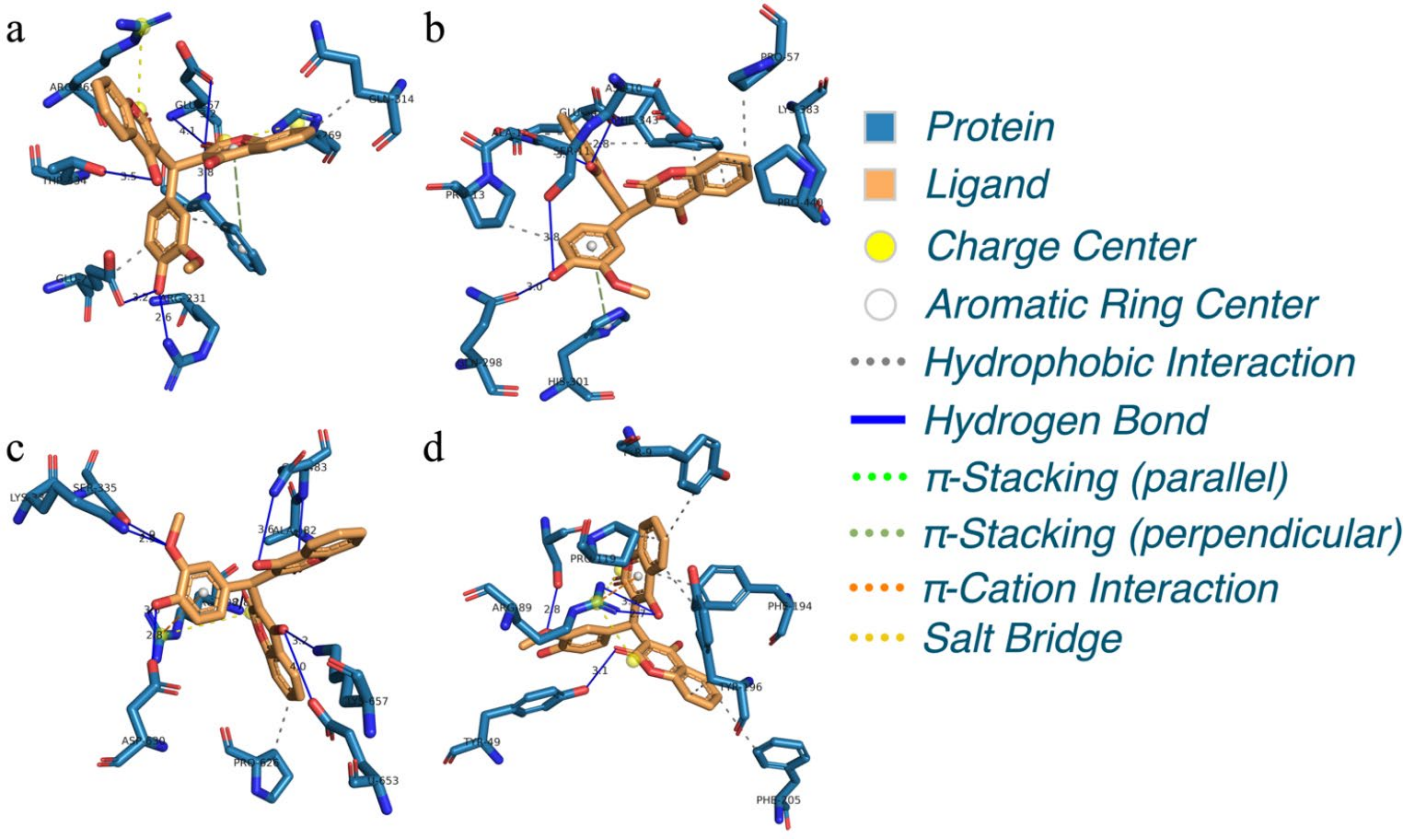
Intermolecular docked poses with lowest binding affinity energies between S1032 and four proteins (3TD3 **(a)**, 4JAS **(b)**, 5BUF **(c)** and 6FJY **(d)**. The labels for contacting residues and the lengths for binding interactions are listed explicitly. The binding interactions are analyzed by PLIP (Adasme et al. 2021)

In the above research, we have revealed that the S1032 has excellent anti-bacteria effect, which could exert protective effect through many different ways, such as influencing the bacterial growth, inhibiting the biofilm formation, and disturbing the bacterial interaction, finally killing the bacteria. However, the detail target of the new compound was still need to be explored. The potential bacterial adaptability and response ability of the S1032 encouraged us to conduct molecular docking simulations, which aimed to identify the potential binding modes and evaluated the interactions between S1032 and target protein structure, by which the underlying mechanism to interfere with multiplication, growth and biofilm formation of *A. baumannii* can be extracted and rationalized. The docking of S1032 and four protein structures resulted in good, acceptable scores and acceptable binding modes, as displayed in Fig. 5. Intermolecular docked poses with lowest binding affinity energies between S1032 and four receptors (3TD3 (a), 4JAS (b), 5BUF (c) and 6FJY (d)). For each of the docking pose, it can be observed that multiple binding interactions were formed in terms of hydrogen bonding interaction (H-Bond), and their binding affinity energies were − 7.42, -9.15, -7.11 and − 7.97 kcal/mol, respectively. 3TD3 stands for the OmpA protein, which an outer membrane protein, response for bacterial biofilm formation, eukaryotic cell infection, antibiotic resistance and immunomodulation (Nie et al. 2020). 4JAS stands for BaeSR and AdeSR two-component regulatory systems (TCSs) significantly influence drug resistance (Lingzhi et al. 2018). 5BUF stands for AroA, which catalyzes the sixth step of the seven-step shikimate pathway, response for bacterial survival. 6FJY stands for CsuE was an important bacterial biofilm formation related protein. Such results suggested that the S1032 formed a good number of hydrogen bonds with active pocket residues of above proteins, indicating higher intermolecular affinity of the formed complexes. These interactions explained the potential mechanism of the new compound S1032.

In order to further understand the binding interactions, the detailed binding information within the binding pockets between S1032 and four proteins were analyzed. Explicitly, for protein 3TD3, S1032 interacted with residues Arg231, Glu229, Glu267, Phe332 and Thr334 (Fig. 5a), moreover, a perpendicular π-stacking interaction is formed with residue Phe332, and two salt bridges are formed with residues Arg265 and His269, for protein 4JAS, the S1032 interacted with residues Asp10, Ser11, Gln298 and Ala12 (Fig. 5b), and a perpendicular π-stacking interaction is formed with residue His301, for protein 5BUF, multiple active residues were involved in the binding interactions with S1032, including Lys334, Ser335, Ala482, Gln483, Arg508, Asp630, Glu653 and Lys657 (Fig. 5c), in addition, a π-cation interaction and a salt bridge interaction are formed with residue Arg508, and for protein 6FJY, the S1032 was seen to interact with residues Ser117, Arg89 and Tyr49 (Fig. 5d), moreover, a π-cation interaction and two salt bridge interactions are formed with residue Arg89. Moreover, for each binding pose, there were multiple active residues that were in the vicinity of the compound, suggesting potential interaction probabilities. Overall, the results from molecular docking simulations suggest that the S1032 exhibits excellent the capability to interfere with multiplication, growth and biofilm formation of *A. baumannii*.

#### Low Cytotoxicity of S1032

**Fig. 6:**
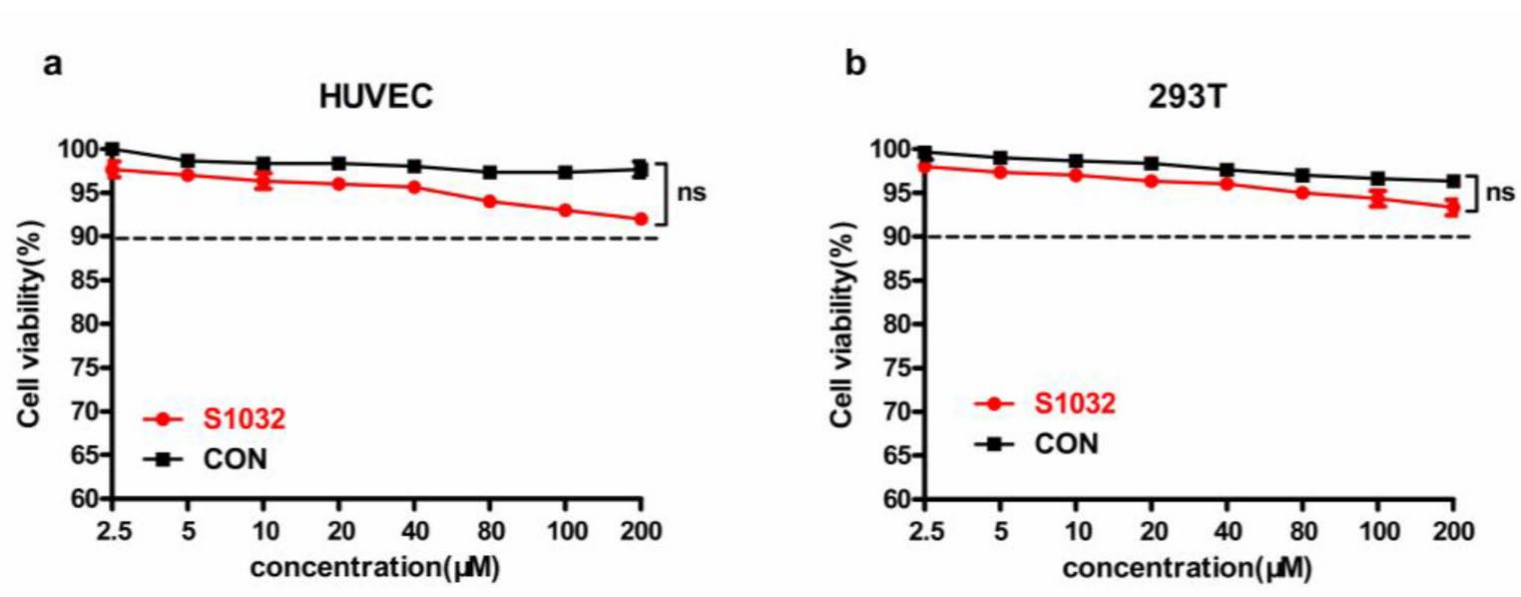
S1032 exhibited low cytotoxicity on normal human cells. The S1032 compound was incubated with HUVEC **(a)** and 293T cells **(b)**, CCK-8 detection kit was used to measure the Cell survival rate of cells

After the pharmacodynamic verification of S1032, its cytotoxicity was then evaluated on HUVEC (Fig. 6a) and 293T (Fig. 6b) cells with CCK-8 detection kit. All the results indicated that the S1032 showed no influence on the viability of the cells, suggesting low cytotoxicity of S1032 during anti-*A. baumannii* infection therapy. This is an excellent advantage of the new compound compared with most commonly used antibiotics in clinic, which may be explained by the special antibacterial mechanism of S1032.

### ADMET Predication of S1032

To study de the pharmacokinetics and toxicity properties of the S1032, ADMET property of this new compound was predicated in this present research. These properties were analyzed via utilizing the swissADME online tool. The ADMET parameters are recorded in Table 5. For S1032 compound, which has moderately soluble property, high GI absorption ability, no CYP inhibition and good druglikeness property. All these results suggested the synthetized compound demonstrated fascinating properties, regarding intestinal adsorption, harmfulness and blood-brain barrier permeability. Therefore, it has the value of further study.

**Table 5:** Pharmacokinetics and toxicity properties of the S1032

**Physicochemical Properties**	
Formula	C_26_H_20_O_8_
Molecular weight	460.43 g/mol
Num. heavy atoms	34
Num. arom. heavy atoms	16
Fraction Csp3	0.15
Num. rotatable bonds	4
Num. H-bond acceptors	8
Num. H-bond donors	3
Molar Refractivity	122.94
PTSA	126.43 Å²
**Lipophilicity**	
Log *P*_o/w_ (iLOGP)	2.86
Log *P*_o/w_ (XLOGP3)	3.13
Log *P*_o/w_ (WLOGP)	3.82
Log *P*_o/w_ (MLOGP)	1.93
Log *P*_o/w_ (SILICOS-IT)	3.04
Consensus Log *P*_o/w_	2.96
**Water Solubility**	
Log *S* (ESOL) Solubility Class	-4.75 8.17e-03 mg/ml ; 1.77e-05 mol/l Moderately soluble
Log *S* (Ali) Solubility Class	-5.45 1.62e-03 mg/ml ; 3.51e-06 mol/l Moderately soluble
Log *S* (SILICOS-IT) Solubility Class	-5.14 3.31e-03 mg/ml ; 7.19e-06 mol/l Moderately soluble
**Pharmacokinetics**	
GI absorption	High
BBB permeant	No
P-gp substrate	No
CYP1A2 inhibitor	No
CYP2C19 inhibitor	No
CYP2C9 inhibitor	No
CYP2D6 inhibitor	No
CYP3A4 inhibitor	No
Log *K*_p_ (skin permeation)	-6.89 cm/s
**Druglikeness**	
Lipinski	Yes; 0 violation
Ghose	Yes
Veber	Yes
Egan	Yes
Muegge	Yes
Bioavailability Score	0.55
**Medicinal Chemistry**	
PAINS	0 alert
Brenk	1 alert: cumarine
Leadlikeness	No; 1 violation: MW > 350
Synthetic accessibility	5.29

The lipid solubility of S1032 is moderate to high, which is beneficial for its transportation across membranes and its drug action. When it comes to its solubility in water, S1032 shows moderate solubility under different prediction methods. This is important for drug absorption and distribution. The pharmacokinetic properties indicate that S1032 has a high gastrointestinal absorption capacity, but it is unlikely to enter the brain through the BBB (blood-brain barrier). Additionally, it is not a substrate of P-gp, which means it is unlikely to be pumped out of cells by the P-gp transporter. At the same time, it is not an inhibitor of major CYP enzymes, indicating that it is unlikely to cause interactions when used with other drugs. In terms of drug similarity and chemical properties, S1032 meets some pharmacophore models and pharmacokinetic screening criteria, such as Lipinski, Ghose, Veber, Egan, and Muegge. Furthermore, it does not have structural features related to PAINS, which may indicate that it is unlikely to cause certain structure-related side effects or adverse reactions. However, it is labeled as a cumarine analog in the Brenk method, which may suggest certain specific biological activities or side effects.

In summary, S1032 exhibits moderate to high lipid solubility and water solubility, and has a high gastrointestinal absorption capacity. In terms of pharmacokinetics, it does not inhibit major CYP enzymes and does not interact with P-gp. From the perspective of drug chemical properties, it has certain drug similarity and potential biological activity.

#### Derivative Optimization Prediction of S1032 Using Reinforcement Learning Progress

**Fig. 7:**
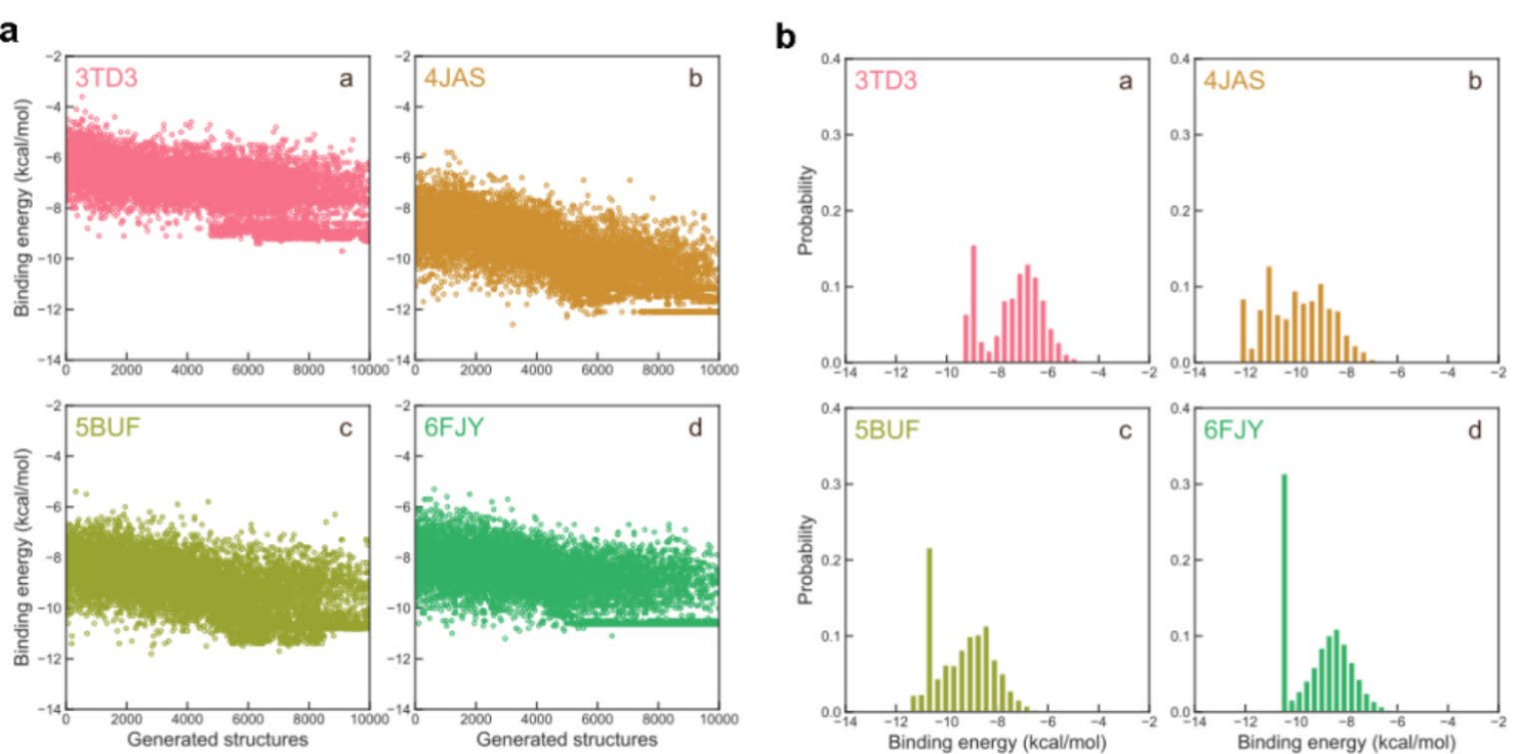
The binding affinity energy between the S1032 and the receptor as a function of reinforcement learning steps (generated structures) for receptors 3TD3 **(a)**, 4JAS **(b)**, 5BUF **(c)**, and 6FJY **(d)**. The corresponding histograms of binding affinity energy between the S1032 and receptors 3TD3 **(e)**, 4JAS **(f)**, 5BUF **(g)**, and 6FJY **(h)**

From the above presented experimental and simulation results, the S1032 has been proven exhibiting excellent bioactivity to interfere with multiple proteins and participate in many processes, such as multiplication, growth and biofilm formation of *A. baumannii*. Thus, the S1032 was used as the training template to investigate possible molecular structures that may have similar or even better bioactivities using the reinforcement learning. As have been mentioned in the above method section, for each of the ligand (S1032) - receptor (3TD3, 4JAS, 5BUF and 6FJY) combination, 20 modifications (actions) are allowed, and up to 10,000 molecule structures are generated and evaluated. The binding affinity energy between the S1032 and the receptor as a function of reinforcement learning steps (optimized structures) are displayed for the four adopted receptors. As one of the indicators for the reinforcement learning progress, a decreasing trend of binding affinity should be observed due to the reinforcement learning nature. In Fig. 7a-d, we can see that all four ligand-receptor combinations exhibit decreasing binding affinity energies with increase in the optimized structures, suggesting a successful reinforcement learning progress from the energetical point of view. Comparing with the training template S1032, for receptor 3TD3, the binding affinity energy is -7.42 kcal/mol, and the binding affinity energies for receptor 3TD3 are from − 3.6 to -9.7 kcal/mol, and about 41% of the optimized structures have a binding affinity energy that is lower than the S1032 (Fig. 7e). For receptor 4JAS, the original binding affinity energy is -9.15 kcal/mol, and the optimized structures are located within the range from − 5.8 to -12.6 kcal/mol, and 70% of the optimized structures have a relatively lower binding affinity energy (Fig. 7f). For receptor 5BUF, the S1032 exert a binding affinity energy of -7.11 kcal/mol, the optimized structures are ranging from to -5.4 to -11.8 kcal/mol, and almost all the generated structures (more than 98%) have lower binding affinity energies than S1032 (Fig. 7g). For receptor 6FJY, the original binding affinity energy is -7.97 kcal/mol, the optimized structures are ranging from − 5.3 to -11.2 kcal/mol, and more than 89% of the optimized structures have a binding affinity energy lower than S1032 (Fig. 7h). Though the above results, we draw two conclusions. First, the binding affinity energy for all receptors decreases with increase in the optimized structures. Second, at least more than 40% of the optimized structures have relatively low binding affinity energy than the training template, suggesting that the reinforcement learning progress is successfully implemented from the energetical perspective.

#### Synthetic Accessibility (SA) Score and Drug-Likeness (QED) Score of New Structures

**Fig. 8:**
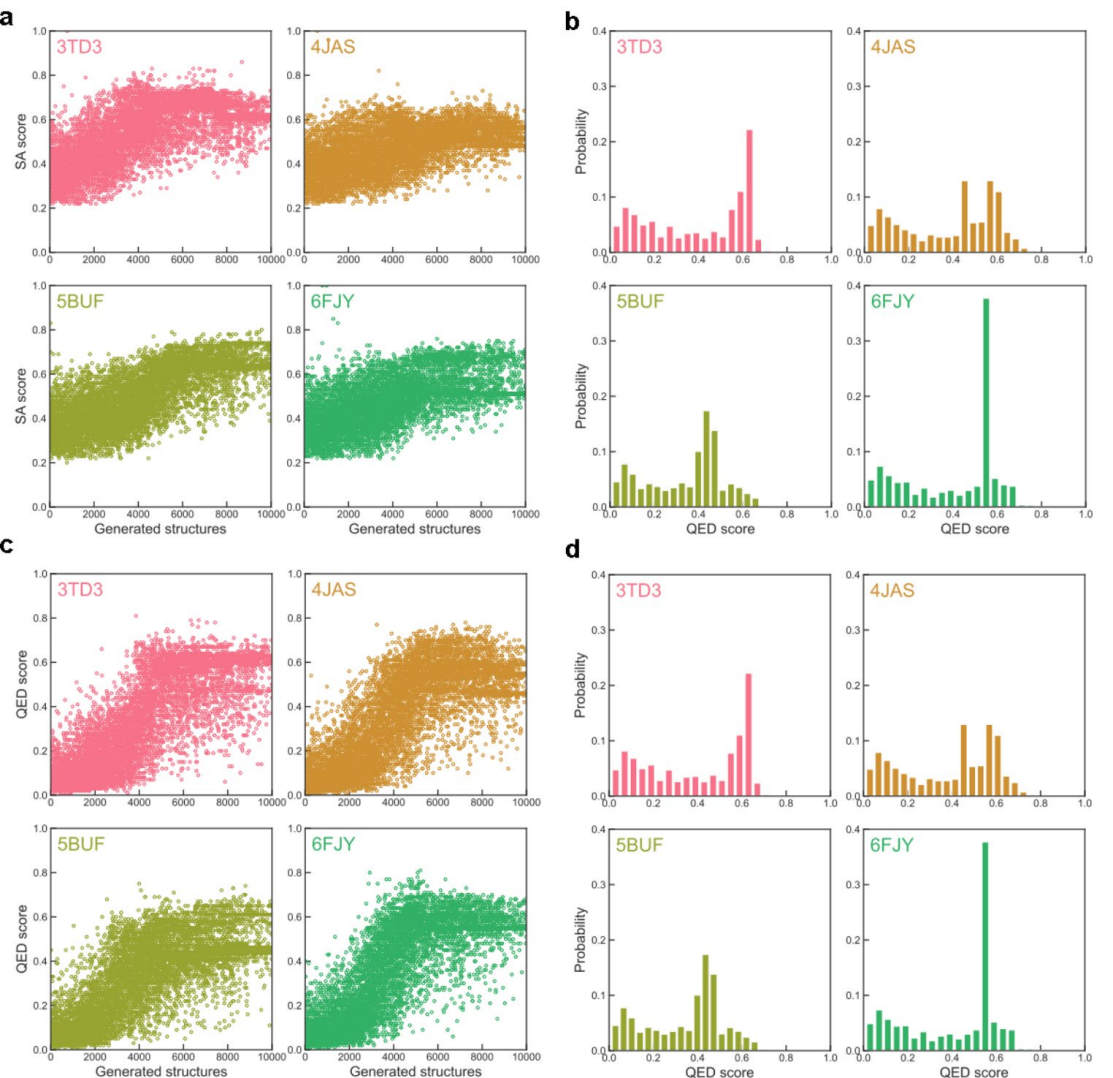
The synthetic accessibility (SA) score for receptors 3TD3, 4JAS, 5BUF, 6FJY **(a)**, and the corresponding histograms **(b).** The quantitative estimate of drug-likeness (QED) score for receptors 3TD3, 4JAS, 5BUF, 6FJY **(c)**, and the corresponding histograms **(d)**

The synthetic accessibility (SA) score that qualifies how easy a given structure can be synthesized comparing to the known structures in the library serves as another qualification characteristics for the reinforcement learning progress, the results for the four adopted receptors are shown in Fig. 8a. Different from the binding affinity energy, the SA score should have an increased trend as a function of the optimization steps, it can be seen that indeed the profiles for all probed receptors have an increasing trend, and basically the SA scores are ranging from 0.2 to 0.8 (Fig. 8b), suggesting a significant number of optimized structures are expected to be easily synthesized.

The final qualification characteristics is the quantitative estimate of drug-likeness (QED) score, which is used to estimated how likely the optimized structure is similar to known drug molecules in the library, the QED scores for the four probed receptors are presented in Fig. 8c. Similar to the results of SA scores, the QED scores also have an increasing trend as a function of the reinforcement learning steps. It can be seen that basically all the QED score profiles are ranging from 0 to 0.8 (Fig. 8d), combining with the above results for binding affinity energy and SA scores, the above results suggest that all reinforcement learning progresses are succeed in generating effective structures based on the given template S1032.

#### New Structures Optimized from S1032 by Reinforcement Learning

**Fig. 9:**
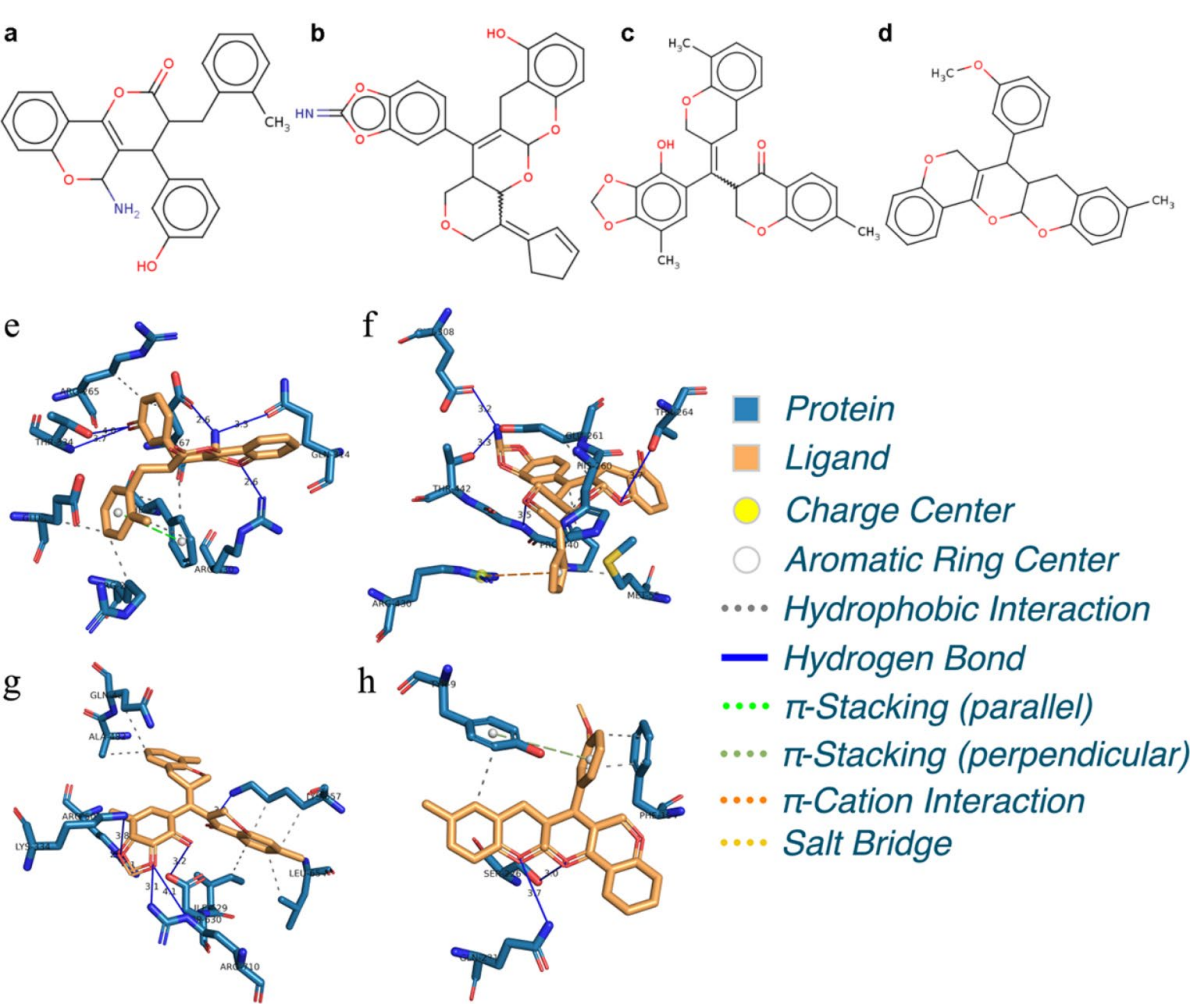
Selected structures that are choosing from 10,000 optimized structures for each of the probed protein **(a-d).** Intermolecular docked poses with lowest binding affinity energies between the optimized structures with respect to four proteins 3TD3 **(e)**, 4JAS **(f)**, 5BUF **(g)** and 6FJY **(h)**. The labels for contacting residues and the lengths for binding interactions are listed explicitly

To further examine the effectiveness of the reinforcement learning, four structures are manually choosing from 10,000 optimized structures for each of the probed receptor (3TD3, 4JAS, 5BUF, and 6FJY), the selected structures are listed in Fig. 9a-d. The binding affinity energies are - 9.3, -11.5, -10.9 and - 10.4 kcal/mol, where are lower than the training template S1032. Further, the SA scores are 0.68, 0.56, 0.68 and 0.69, and the QED scores are 0.63, 0.55, 0.55, and 0.56, respectively.

Although an energy minimization procedure has been conducted during the reinforcement learning progress for each of the optimized structure, however, such an energy minimization proved by the Open Babel serves as an early estimation of the structure on the potential surface. Thus, the four selected structure have been further optimized by the GAFF force field, and 50 binding poses for each of the structure have been estimated again through AutoDock. The intermolecular docked poses with lowest binding affinity energies between the optimized structures with respect to four receptors are displayed in Fig. 9e-h, the corresponding binding affinity energies are - 10.56, -10.65, -7.58 and - 7.75 kcal/mol, these binding affinity energies are seen to be better or comparable with the training template.

Further, it is observed that for protein 3TD3, the selected structure (Fig. 9e) forms four binding interactions with residues Glu267, Gln314, Arg330 and Thr334, in addition, one parallel π-stacking interaction is formed with residue Phe332. For protein 4JAS, the selected structure (Fig. 9f) is seen to form four hydrogen bonds with residues Thr264, Glu308, Thr442 and Gly441, and a π-cation interaction is formed with residue Arg430. For protein 5BUF, the selected structure (Fig. 9g) has formed seven hydrogen bonds with residues Lys657, Lys334, Arg508, Arg710 and Asp630. And for protein 6FJY, there are two binding interactions are formed between the selected structure (Fig. 9h) and residues Ser226 and Gln231, and a perpendicular π-stacking interaction is formed with residue Tyr9. The above results demonstrated that the optimized structures from reinforcement learning progress not only have better or comparable qualification characteristics comparing to their training template, the optimized structure indeed exhibit excellent bioactivities with the probed protein.

## Conclusion

As a multi-drug resistant and invasive pathogen, *A. baumannii* is one of the major causes of nosocomial infections in the current healthcare system. It is considered as the leading pathogen of pneumonia, sepsis, meningitis, urinary tract and wound infections, and is associated with high mortality rates in clinic (Ayoub Moubareck et al. 2020). *A. baumannii* has a remarkable ability to acquire or up-regulate various resistance determinants, which making the occurrences of extensively drug-resistant (XDR) and pandrug-resistant (PDR) isolates. Besides, *A. baumannii* also has the strong ability to form bacterial biofilm to avoid the antibiotic exposure. Now, *A. baumannii* has been assigned as a critical priority pathogen with great threat to human health by World Health Organization (WHO). Thus, new antibiotics with novel target and antibacterial mechanism are urgently needed, it is also a hot spot in the field of infection disease treatment.

Recently, the coumarins exhibit a plethora of biological activities, such as anti-inflammatory and anti-tumor effect. In 2016, Jyoti Dandriyal has revealed that the C-4 substituted coumarin derivatives showed excellent anti-cancer activity (Abdelaziz et al. 2022). The Yang Hu revealed the also showed coumarin derivatives were considered as a promising lead compound for the development of commercial drugs (Hu et al. 2019). In this present research, the coumarin derivatives (1–5) and derivatives (6–10) were synthesized, and their application values on the *A. baumannii* infection was evaluated in vivo and in vitro. The results of the MIC values and the *A. baumannii* growth curves suggested the coumarin derivatives have much stronger anti-bacterial activity than the dihydropyran derivatives. While, among the coumarin derivatives, compound **1** (S1032) exhibited the strongest biological activity. Even though the compound **2** has similar structure with the S1032, but its anti-bacterial activity was much weaker than the S1032. According to this, we speculate that the substituent of the S1032 could increase the polarity of the compound, which then increase the concentration of the compound in the plasma. Besides, the crystal structure of S1032 made it bind with the target more stronger than the compound **2**. The S1032 was then used for anti-*A. baumannii* infection evaluation in vivo. After treated with the S1032 with the concentration of 1, 2, 5 mg/kg, the survival rate of *A. baumannii* infected mice and bacterial load in the lung tissue was also reduced in a dose dependent manner. For the bacterial biofilm formation, the S1032 could also reduce the bacterial biofilm formation and the related gene expression in a dose dependent manner. All the data in this this research showed that the S1032 may be an excellent candidate for the *A. baumannii* infection treatment through inhibiting the bacterial biofilm formation and inducing the ROS production.

Moreover, in order to unveil the underlying mechanisms of the bacterial adaptability and response ability of the S1032, molecular docking simulation has been performed to study the capability of the compound to interfere with multiplication, growth and biofilm formation related protein of *A. baumannii*, such as outer membrane protein OmpA, TCSs protein BaeSR and AdeSR, shikimate pathway protein AroA and bacterial biofilm formation related protein CsuE. This predication results suggested the S1032 could bind with a variety of proteins with different functions, then interferes with many physiological functions of *A. baumannii*. Next, based on the molecular docking results and ADMET predication, more structures with higher affinity with the targets were predicated with reinforcement learning progress using S1032 as the training template, and more than 40% of the optimized structures have lower binding affinity energies than the S1032. Four selected structures have been further estimated and exhibit excellent bioactivities, indicating that the reinforcement learning progress we developed is a powerful tool for designing and optimizing novel drug molecules based on a given template. However, the biological activities of these compounds still need to be evaluated in the further research.

In summary, this is a profoundly significant study. Targeting the difficult-to-treat pathogen *A. baumannii*, this study offers us a new perspective for designing novel anti-infective drugs. By synthesizing coumarin derivatives with anti-*A. baumannii* activity and screening them, we can evaluate their biological effects both in vitro and in vivo. This research approach not only helps us understand the mechanism of these compounds, but also aids in a better comprehension of the mechanism of resistance of *A. baumannii*.

Moreover, exploring the action mechanisms and potential targets of these novel compounds can provide valuable information for future drug design and optimization. This not only facilitates the development of more effective anti-infective drugs, but also helps us better understand the life processes of the pathogen, thus offering new insights for future treatment strategies. Overall, this study provides valuable theoretical and experimental data for the development of anti-infective drugs and offers new hope for solving the global problem of *A. baumannii* infections.

## Data Availability

The data used to support the findings of this study are included within the article.
